# Antibacterial activity and biofilm inhibition of a novel bioactive glass coating (F18) on titanium discs

**DOI:** 10.1590/0103-644020267059

**Published:** 2026-10-19

**Authors:** Ísis de Fátima Balderrama, Karoline Bernardes do Nascimento, Clovis Wesley Oliveira de Souza, Marina Trevelin Souza, Edgar Dutra Zanotto, Elcio Marcantonio

**Affiliations:** 1 Department of Diagnosis and Surgery, São Paulo State University, Araraquara School of Dentistry, UNESP, Araraquara, São Paulo, Brazil; 2 Microbiology and Parasitology Laboratory, Department of Morphology and Pathology, Federal University of São Carlos, São Paulo, Brazil; 3 Vitreous Materials Laboratory, Department of Materials Engineering, Federal University of Sao Carlos, São Carlos, São Paulo, Brazil; 4 Vetra Ltda., Ribeirao Preto, São Paulo, Brazil

**Keywords:** Titanium alloy, bioactive glass, antimicrobial, anti-biofilm, biomaterial

## Abstract

This study aimed to investigate the anti-biofilm and bactericidal activity of biofunctionalized titanium with either nanohydroxyapatite or F18 bioactive glass against Staphylococcus aureus biofilms. For that, discs (6.0mm diameter and 3.0 mm thickness) with different surface treatments were studied and divided into three groups: 1) machined titanium (M); 2) coated with Nano-hydroxyapatite (Nano-Ha); 3) Biofunctionalized with F18 bioactive glass (BSF18). Surface characterization was investigated through energy-dispersive spectroscopy, scanning electron microscopy, atomic force microscopy, and bioactivity assays. Antibacterial activity was investigated according to the JIS Z 2801:2010 standard and ISO 22196:2011. The samples were evaluated for UFC/mL, biofilm morphology, and confocal laser scanning microscopy was performed after 24 h. The results showed that BSF18 biofunctionalized discs significantly reduced viable bacterial adhesion compared with Nano-Ha and M groups (3.93±0.14; 5.68±0.26; and 5.57±0.16; p<0.0001; respectively). The morphology analysis revealed a smaller number of adhered bacterial cells for BSF18 samples when compared to the M and Nano-HA, additionally, the biofunctionalization of titanium discs with F18 bioactive glass was effective in inhibiting the growth of Staphylococcus aureus biofilm after direct contact for 24 hours analyzed by confocal laser microscopy which demonstrated greater values for dead cells and lesser live cells for BSF18 surface when compared to machined and Nano-HA (p<0.05). F18 bioactive glass can be a promising biomaterial for preventing and controlling infection by S. aureus on titanium implants. It could be a valid infection-preventing strategy for implant dentistry clinical practice.

## Resumo

Este estudo teve como objetivo investigar a atividade de antibiofilme e bactericida do titânio biofuncionalizado com nanohidroxiapatita ou vidro bioativo F18 contra biofilmes de Staphylococcus aureus. Para isto, discos (6,0 mm de diâmetro e 3,0 mm de espessura) com diferentes tratamentos de superfície foram estudados e divididos em três grupos: 1) Titânio usinado (M); 2) Revestido com nanohidroxiapatita (Nano-Ha); 3) Biofuncionalizado com vidro bioativo F18 (BSF18). A caracterização da superfície foi investigada por meio de espectroscopia de dispersão de energia, microscopia eletrônica de varredura, microscopia de força atômica e ensaios de bioatividade. A atividade antibacteriana foi investigada de acordo com a norma JIS Z 2801:2010 e ISO 22196:2011. As amostras foram avaliadas quanto à UFC/mL, morfologia do biofilme, e a microscopia confocal de varredura a laser foi realizada após 24 horas. Os resultados mostraram que os discos biofuncionalizados com BSF18 reduziram significativamente a quantidade de bactérias viáveis aderidas em comparação com os grupos Nano-Ha e M (3,93±0,14; 5,68±0,26 e 5,57±0,16; p<0,0001; respectivamente). A análise morfológica revelou um menor número de células bacterianas aderidas nas amostras BSF18 em comparação com M e Nano-Ha. Além disso, a biofuncionalização dos discos de titânio com vidro bioativo F18 foi eficaz na inibição do crescimento de biofilme de Staphylococcus aureus após 24 horas de contato direto, analisada por microscopia confocal a laser, que demonstrou maiores valores de células mortas e menor quantidade de células vivas na superfície BSF18 em comparação com o titânio usinado e Nano-Ha (p<0,05). O vidro bioativo F18 pode ser um biomaterial promissor para a prevenção e controle de infecções por S. aureus em implantes de titânio, assim como, uma pode ser uma estratégia válida de prevenção de infecções para a prática clínica da implantodontia.

**Figure:**
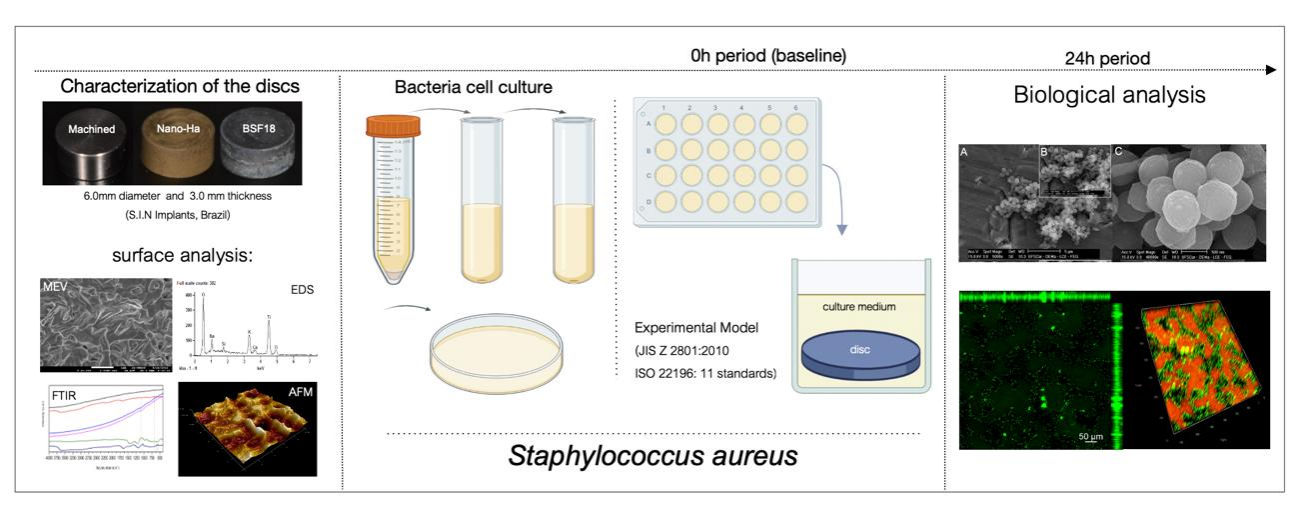

## Introduction

Peri-implantitis is a disease characterized by inflammation of the tissue surrounding dental implants, which culminates in loss of bone support and has become the main cause of implant failure ^1^. The prevalence of peri-implant mucositis is reported to range from 19 to 65%, and for peri-implantitis from 1 to 47%, demonstrating a weighted mean prevalence of 43% and 22% ^2^. Microbial colonization and biofilm formation on implant surfaces are important factor that leads to peri-implantitis, and they are heavily influenced by the implants’ surface roughness and their general physicochemical properties ^3^ ^,^ ^4^. Although the roughness of dental implants is correlated with increased bone-to-implant contact and faster osseointegration, it is also prone to promote biofilm accumulation and hinder the decontamination process ^5^. Peri-implantitis infections are characterized by increased numbers of numerous and diverse bacterial species, including S. aureus ^6^ ^,^ ^7^. So, several bacteria are involved in peri-implantitis evolution, but one of the most important is *Staphylococcus aureus* (*S. aureus)* ^8^. Studies have demonstrated a high affinity of this Gram-positive bacterium for titanium dental implants, as an early colonizer and related to biofilm formation on their surfaces ^9^ ^,^ ^10^. 

Bioglasses are known to strongly bond to bone due to their high bioactivity, especially Class A bioactive glasses. These materials can rapidly form a hydroxycarbonate apatite layer on their surface, which allows them, among other mechanisms, to chemically bond to the host tissue ^11^. Concomitantly, some bioglass compositions demonstrated a high antimicrobial effect, inhibiting bacterial growth through osmotic and acid-base imbalance ^12^. So, bioactive glasses can be a promising alternative to control and prevent peri-implantitis. It was reported that at the first moment of contact of the bioactive glass and the surrounding body fluids, there is an increase in pH, resulting in an alkaline microenvironment. The release of sodium, silica, calcium, and phosphate from the bioactive glass surface increases salt concentration and osmotic pressure ^13^. This ion-leaching process is considered an important feature of the antibacterial activity of bioglasses ^14^. Although the mechanisms involved in the antibacterial activity of bioglasses are still under investigation, they are probably based on a combination of several factors, including the high pH and osmotic effects caused by the dissolution of the glass network and network-modifying ions ^15^ ^,^ ^16^. A novel bioactive glass composition named F18 belongs to the system SiO2-Na2O-K2O-CaO-MgO-P2O5 and shows greater workability than other bioactive glasses, while maintaining high bioactivity and broad bactericidal activity ^17^. 

Previous studies have shown that F18 Bioglass, both in powder and fiber forms, was an efficient bactericidal agent against several strains, such as *Staphylococcus aureus*, *Staphylococcus epidermidis*, *Escherichia coli,* and *Pseudomonas aeruginosa* ^17^. Additionally, surfaces coated with F18 reduced *S. aureus* growth ^18^. Titanium coated with F18 bioactive glass, aimed at applications in the orthopedic field, presented lower cell adhesion (S. epidermidis and P. aeruginosa) after 2, 4, and 8 hours when compared to plain titanium ^19^. These results potentially demonstrate that F18 can be an alternative material for controlling and treating infections. Titanium and other metals are artificial materials and have no biofunctionality; however, to insert biofunctionality into these materials, surface modification is necessary. Biofunctionality cannot be inserted during their manufacturing process, for example, melting, casting, forging, and heat treatment ^19^. 

The surface modification consists of changing the material surface composition through its composition, structure, and morphology, allowing the bulk mechanical properties to remain intact ^20^. Biofunctionalization is one particular form of surface modification involving the immobilization of biomolecules such as proteins, peptides, polysaccharides, or bioactive drugs/materials with the same purpose ^21^.The biofunctionalization has mainly two objectives: the enhancement and/or the inhibition of protein adsorption, and the improvement of cell adhesion ^20^. 

F18 bioactive glass coating of implants can be an interesting approach for Implant Dentistry clinical applications since a preclinical study with dental implants coated with bioglass showed better osseointegration (bone-implant contact; BIC) in the first 2 weeks of implantation when compared to the control group (advertised as the best surface treatment available in the market by the time the study was conducted)^22^. In this study, the sandblasted and dual acid-etched surface was used as a control, and the F18 biofunctionalized implants (BSF18) demonstrated greater values of BIC and bone density (BD) at 2 weeks post-implantation (25.5% and 18.0% of BIC, and 40.7% and 27.2% of BD, respectively). Therefore, new approaches for clinical applications are based on the investigation and development of new implant coatings, and this preliminary study aimed to investigate the anti-biofilm and bactericidal effect of BSF18 biofunctionalization on titanium surfaces against *Staphylococcus aureus* biofilm. 

## Methodology

### Bioactive glass and disc surface preparation

Titanium discs with a 6 mm diameter and 3 mm thickness were supplied by S.I.N. Implants (São Paulo, SP, Brazil). In this study, three types of surfaces were investigated: as-prepared (machined M - control group), Nano-hydroxyapatite blasted (Nano-Ha), and nano-HA + F18 bioglass biofunctionalized (Nano-BSF18). The machined surface was titanium commercially pure (grade IV). The Nano-Ha surface was prepared according to the HA nanocrystalline synthesis method using the Pomimic HA^nano^. This step was performed by the titanium disc provider. Briefly, the discs were dipped into a stable particle suspension containing 10 nm in diameter HA particles, followed by a heat treatment at 550 ⁰C for 5 minutes in a nitrogen atmosphere. Importantly, the HA calcium-to-phosphorus ratio used was 1.67. This was a surfactant-mediated process that allowed better control of the chemical composition of the coating, which has a thickness of ≤150 nm ^23^. Nano-Ha discs were then biofunctionalized with bioglass particles of F18 (SiO_2_-Na_2_O-K_2_O-MgO-CaO-P_2_O_5_ system) using the pneumatic atomization technique to obtain biofunctionalized bioactive glass discs (Nano-BSF18). 

Briefly, the analytical-grade oxides were mixed and melted in a platinum crucible at 1450 °C for 3 hours and quenched by splat cooling. Powdered samples were prepared by grinding, and the coating was performed according to BR10 2014 003817 5 patent ^17^. All tests were performed independently in triplicate for each sample.

### Ultra-high-resolution scanning electron microscopy with field emission (SEM-FEG) and energy dispersive X-ray spectroscopy (EDS) analysis

The morphological characterization of the studied surfaces was analyzed by Scanning Electron Microscopy (SEM; JSM-7500F, JEOL, PC-SEM operation, Tokyo, Japan) coupled with secondary electron detectors, backscattered and chemical analysis (EDS-Energy Dispersive X-ray spectroscopy, Thermo Scientific brand, Ultra Dry model, with NSS 2.3 operating and an accelerating voltage of 10.0 kV) was used to evaluate the chemical elements on different surfaces (% atomic). 

### Atomic force microscopy

Nanoscale roughness was evaluated by atomic force microscopy (AFM) for all tested groups (n=3/Nano-Ha and n=3/BSF18). In this analysis, the AFM tip acts as a ″nano profilometer″, being able to generate information on the micro- and nano-dimensional aspects of the surface (x, y, and z-axis). Images were acquired by the microscope (Nanoscope V model, Bruker, Massachusetts, USA) with a frequency of 50-90 kHz and a scan rate of 0.330 Hz with multiple modes of operation and formation and imaging. The equipment worked under an intermittent contact model with ScanAsyst software. Images were taken from disc samples in 3 different regions for each sample (border, middle, and opposite border). Scans of 10 × 10× µm were taken to check homogeneity. The analyzed roughness parameters were R_a_ (arithmetic average roughness), R_q_ (root mean square roughness), and R_max_ (maximum height of the profile).

### Bioactivity assay

To analyze the effect of F18 on the surface of the Ti disc and identify any changes in its bioactivity, tests evaluating the samples’ apatite-forming ability were performed, following and adapting the ISO 23317:14 recommendations ^24^. Samples were soaked in 25 mL of Simulated body fluid (SBF) solution for 0 h (baseline) and 24 hours. No solution changes were made during the test, followed by Fourier Transform Infrared Spectroscopy (FTIR) analysis, ranging from 4000 to 400 cm^-1^ (Perkin Elmer, model Spectrum GX-DE). These tests aimed to monitor and measure the degradation of the bioactive glass and the speed of formation of the hydroxycarbonate apatite layer (HCA) on the specimen surface, as well as to evaluate the bioactivity of Nano-Ha samples.

### Antimicrobial tests

### Bacterial strain and inoculum preparation

Antibacterial activity tests were conducted using the methodology proposed by JIS (Japanese Industrial Standard) Z 2801:2010 and ISO 22196:11. The bacterial strain *Staphylococcus aureus* (ATCC 25923) was used for the inoculum preparation. The bacterial strain was transferred from its stock culture (-20⁰C) to the medium and incubated for 24 hours at 36⁰C ± 1⁰C. Inoculation was standardized by measuring spectrophotometric absorbance at 600 nm, reaching an absorbance value of approximately 10^8^ Colony Forming Units per milliliter (CFU/mL). Standardized suspensions were diluted to obtain a 10^6^ CFU/mL concentration required for the initial inoculum. All tests were performed in triplicate for each sample.

### Quantification of viable bacteria by colony-forming unit (CFU) analysis

For the analysis of the quantification and possible reduction of *S. aureus* biofilm, 1 mL of the standardized bacterial inoculum was placed on the surface of the discs and incubated at 36⁰C ± 1⁰C for 24 hours. After the incubation, the samples were washed with PBS. The bacterial biofilms were disaggregated, and serial dilutions of 10 to 10.000 times were obtained. Spreading was performed according to the Spread Plate method. The counting was performed using mechanical counting of bacteria (cp602 machine, Phoenix Luferco, Araraquara, Sao Paulo, Brazil).

### Biofilm morphology

To evaluate biofilm morphology, all discs were washed with PBS and fixed in a 2.5% glutaraldehyde solution, followed by gradual dehydration with ethyl alcohol and followed by gold coating for SEM images. All samples were submitted to SEM images performed by High-Resolution Scanning Electron Microscopy (FEG-SEM; JEOL) equipped with an EDS accessory (energy dispersion X-ray spectroscopy). To analyze the morphology, the alteration of the cell and its structure will be considered

### Confocal laser scanning microscopy

Following the protocol used by Tavares and co-authors ^25^, the biofilm thickness and bacterial cell viability were evaluated after staining with Live/Dead BacLight TM Bacterial Viability staining assay (Invitrogen, Carlsbad, CA, USA) according to the manufacturer’s instructions. This analysis is based on a combination of fluorescent reagents able to distinguish live bacteria^13^ with intact plasma membranes from dead bacteria (red) with compromised membranes. After PBS washing, biofilms were analyzed with an LSM 800 confocal laser-scanning microscope (Zeiss, Jena, Germany). Images were taken through a 10x dry (EC Plan-Neofluar NA 0.3 air) objective. The excitation wavelength of 488 nm (Ar laser) and 561 nm (HeNe laser) were employed to visualize the distribution of live and dead bacteria. 

A quantification evaluation of the percentage of live and dead bacteria adhered to surfaces was analyzed; therefore, the images were converted to 8 bits by the software ImageJ® (NIH, EUA) and evaluated by a blind examiner according to the calculation of total area and presence of live or dead bacteria ^26^. 

### Statistical analyses

Group comparisons were performed using the Shapiro-Wilk test to assess normality. The data were analyzed by ANOVA with a 95% confidence level, followed by a Tukey test using GraphPad Prism software. The CFU/mL counts were transformed into log10 and subjected to one-way analysis of variance (ANOVA) with Tukey’s multiple comparisons tests (p<0.05). The counting (n) of live and dead cells analyzed by ImageJ software was evaluated by two-way analysis of variance (ANOVA) with Tukey’s multiple comparisons tests (p<0.05). The data presented were plotted as the mean and standard deviation (SD). For SEM and confocal analysis, experiments were performed three times in triplicate to ensure reproducibility. 

## Results

### Scanning Electron Microscopy and Chemical Characterization

The SEM images of the Nano-HA and Nano-BSF18 samples showed clear differences in surface morphology compared to the machined (M) discs. Figure 1 shows representative images at 20,000× magnification of machined (A), Nano-HA (B), and Nano-BSF18 (C). The chemical analysis by EDS was carried out for all samples, and the chemical elements were assessed. Titanium was detected for all groups; oxygen and calcium for Nano-HA and BSF18; phosphorus only for Nano-HA; potassium and silicon only for BSF18. Nano-Ha showed the presence of a percentage of chemical elements (% atom), such as titanium (64,79%), oxygen (31,94%), calcium (1,99%), and phosphorus (1,28%). On the other hand, BSF18 showed the presence of titanium (27,91%); oxygen (58,99%); potassium (7,65%); sodium (3,18%); calcium (1,55%), and silicon (0,73%). Machined discs showed only the presence of the titanium (100%) chemical element. These chemical analyses demonstrated the exact presence of chemical elements on each surface (Figure 2). 

**Figure 1.**
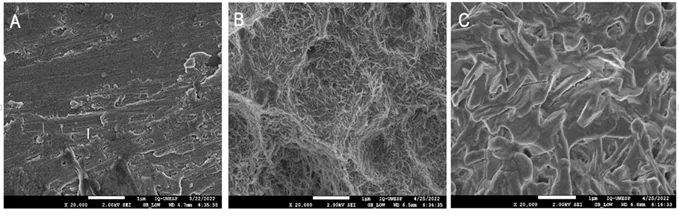
Scanning electron microscopy images with 20.000x magnification. A. Machined surface; B. Nano-hydroxyapatite surface; C. BSF18 Surface.

**Figure 2:**
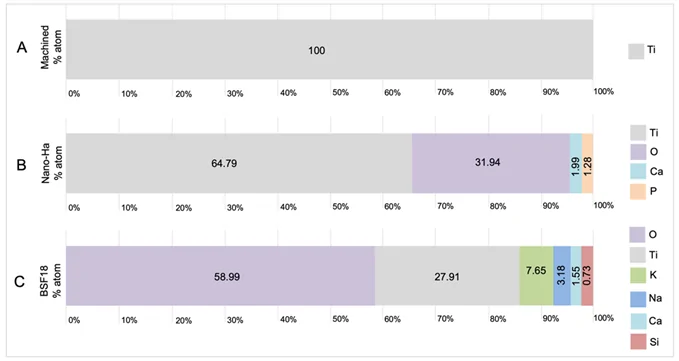
The values represent the percentage (%) of atoms for Machined (A); Nano-hydroxyapatite (Nano-Ha) and bioactive glass (BSF18) surfaces.

### Atomic Force Microscopy

Atomic force micrographs showed that there is a significant difference among the evaluated surfaces. The nano-scaled topography promoted by blasting HA (Figure 3-B) and coated with bioactive glass F18 (Figure 3-C) was confirmed by AFM. Table 1 shows the values related to Ra, Rq, and Rmax, presenting the statistical difference between the surfaces. The machined samples, as expected, showed lower values for all parameters when compared to Nano-Ha and Nano-BSF18 (Table 1). 

**Figure 3:**
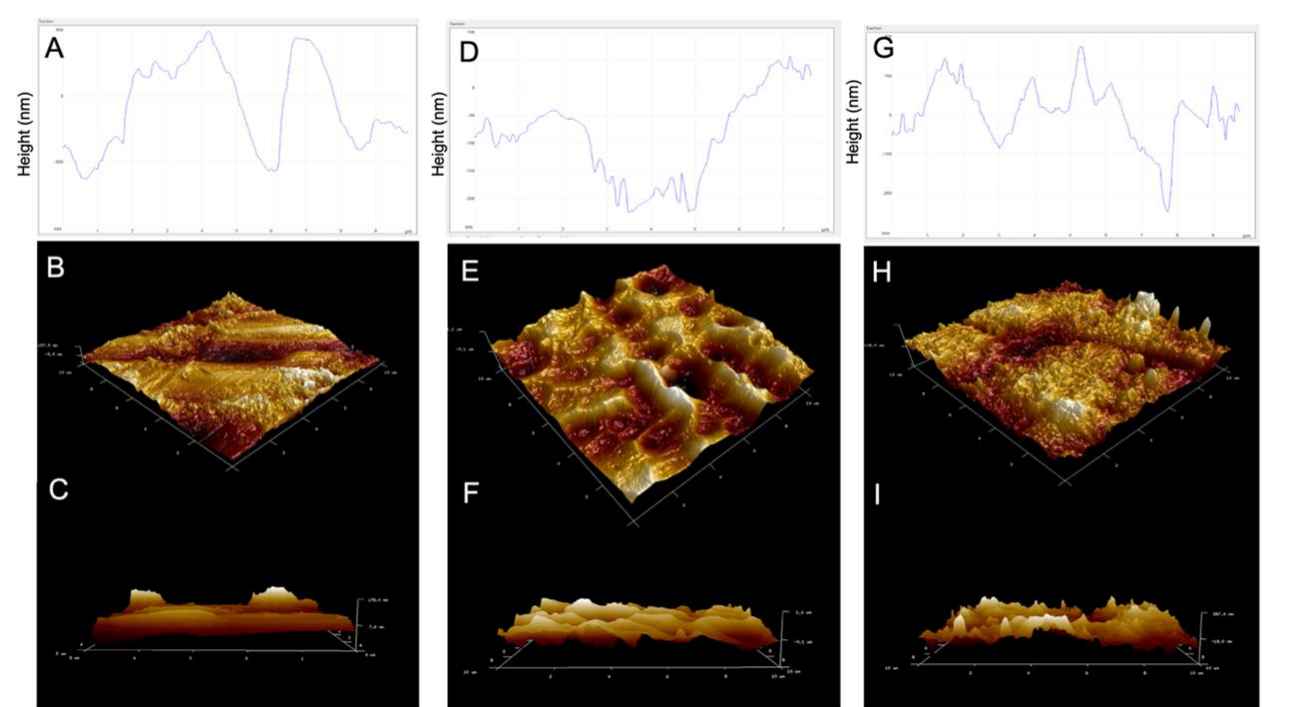
Fourier Transform Infrared Spectroscopy analysis to identify the formation of the hydroxycarbonate apatite layer. A. BSF18 Surface after 0 and 24 hours soaking in SBF solution. B. Comparison of all groups investigated.

**Table 1:** atomic force microscopy values (Mean±Standard Deviation) for all the studied groups; Kruskal Wallis test (p<0.05). Different letters in the same column indicate a statistical difference between surfaces, for Ra (p=0.02); Rq (p=0.03); Rmax (p=0.02).

Sample	R_a_ (nm)	R_q_ (nm)	R_max_ (nm)
Machined	5.75±1.0^a^	7.79±1.18^a^	44.23±16.4^a^
Nano-Ha	51.7±13.8^b^	68.93±11.1^b^	451±57.7^b^
BSF18	46.33±4.5^b^	58.86±4.6^b^	376.33±11.9^b^
*p* value	*0.02*	*0.03*	*0.02*

### Bioactivity

Figure 4 illustrates the obtained curves for 0 and 24 hours of immersion in SBF-K9 solution for machined, Nano-Ha, and Nano-BSF18 samples. The only sample that precipitated HCA after 24 h immersion in SBF-K9 was the BSF18 (Figure 4-A). The dotted lines in Figure 4-A show the most characteristic peaks for HCA formation: 560 and 602 cm^-1^ [19]. For machined and Nano-HA, no significant changes were observed. In Figure 4-B, all the curves are shown in the same graph for better visualization and comparison between the groups

**Figure 4:**
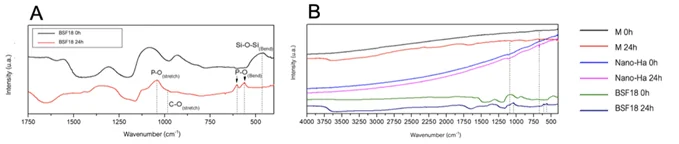
Fourier Transform Infrared Spectroscopy analysis to identify the formation of the hydroxycarbonate apatite layer. A. BSF18 Surface after 0 and 24 hours soaking in SBF solution. B. Comparison of all groups investigated.

### Quantification of viable bacteria by colony-forming unit (CFU) analysis

The CFU/mL count showed that all bacterial strains formed amounts of mature biofilm on the surface discs; however, a higher CFU was detected for the Nano-Ha and Machined surfaces when compared to BSF18. Figure 5 shows no statistical difference for Machined vs. Nano-Ha (p>0.05); however, both Machined and Nano-Ha showed a statistical difference when compared to BSF18 (5,57±0,16; 5,68±0,26 and 3,93±0,14, respectively; p<0.0001). As the antibacterial activity (or the reduction of viable bacteria) is calculated by the equation: R= (U_t_ - U_0_) - (A_t_ - U_o_), being R the antibacterial activity, Uo the average of logarithm numbers of viable bacteria immediately after inoculation on untreated test pieces; Ut the average of logarithm numbers of viable bacteria after inoculation on untreated test pieces after 24h and At the average of logarithm numbers of viable bacteria after inoculation on treated test pieces after 24h, we can have that the bacterial reduction was of 1.64 for BSF18 and bacterial growth of 0.11 for Nano-Ha compared to the machined samples.

**Figure 5:**
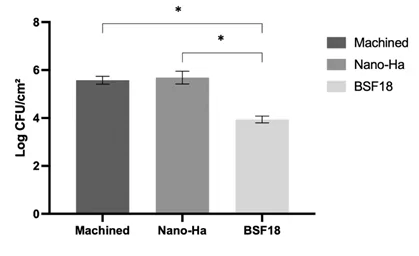
No statistical difference for Machined vs. Nano-Ha (p>0.05) was found; however, both Machined and Nano-Ha showed a statistical difference when compared to BSF18 (5,57±0,16; 5,68±0,26 and 3,93±0,14, respectively; p<0.0001). One-way ANOVA test with Tukey’s comparisons**.**

### Biofilm morphology

The morphology of the attached bacteria was determined by SEM analysis in parallel to CFU counting. The 24-hour SEM images of S. *aureus* biofilm showed distinct adhesion behavior between the evaluated surfaces. It can be observed that the cells that were in contact with F18 bioactive glass showed significantly less agglomeration or clusters and lower amounts of cells when compared to the other groups (Figure 6- G, H, and I), corroborating with the quantitative results. As shown in Figure 6, this analysis revealed a remarkable difference in *S.aureus* cells adhered to different surfaces. Bacterial cells on the BSF18 surface were opaque, round, with a smooth surface, and with clear division septa (Figure 6-G, H, I). Dividing microorganisms were very frequent. For the Machined and Nano-Ha specimens, it was often observed that several bacterial cells clustered together and were covered by a dense and gray layer resembling a blanket. Individual cells were tightly associated with each other and connected by junctions. In addition, in most instances, there was a halo surrounding the bacteria at the contact interface between the bacterial cells and the Ti surface. 

**Figure 6:**
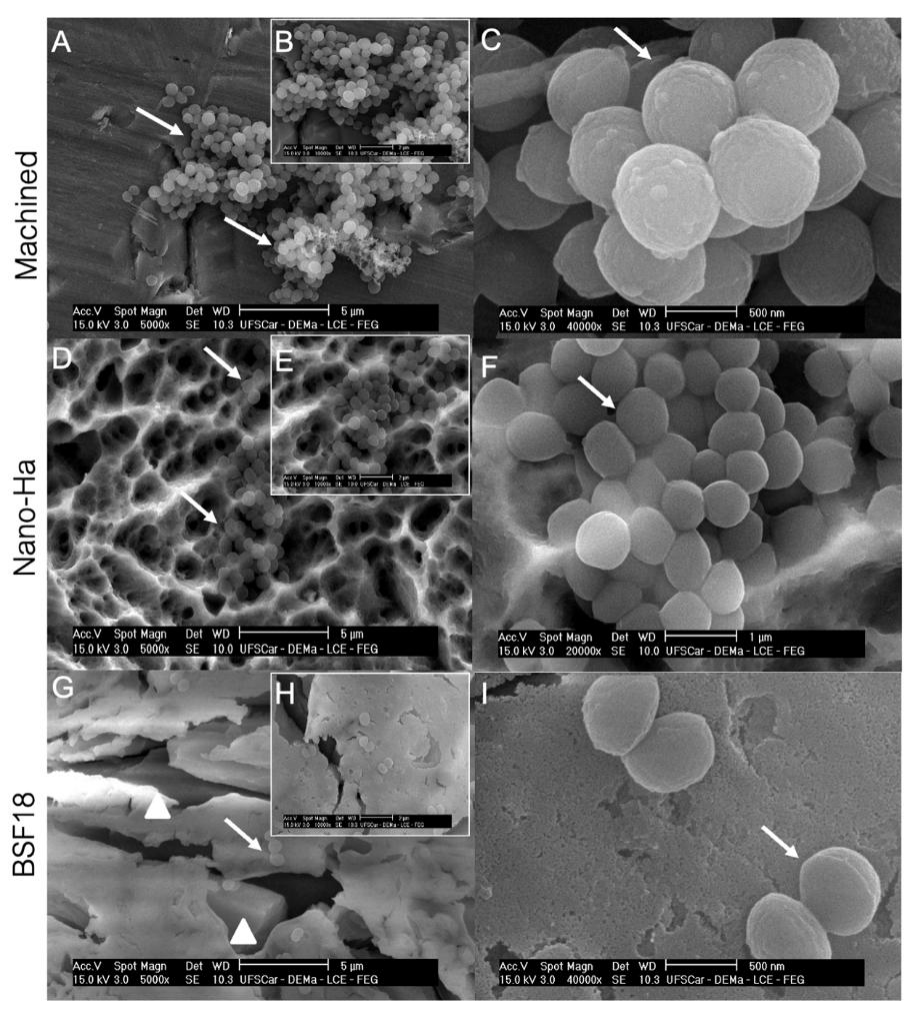
Scanning electron microscopy of the formed biofilms after 24 hours in contact with A, B, C: Machined*.* D, E, and F: Nano-hydroxyapatite. G, H, and I: BSF18 samples. Different magnifications are presented: 5.000x; 10.000x and 20.000x. Arrows indicate the adhered bacteria, and the head arrows indicate the BSF18 glass surface.

### Confocal laser scanning microscopy

The quantification of live and dead cells on the surface of the material was evaluated only for the green (live) and red (dead) staining with 50 µm of thickness. The distribution of stained bacteria and biofilm thickness under confocal analysis confirmed the CFU/mL results previously obtained, with an expressive number of dead bacteria only for the BSF18 surface when compared to Machined and Nano-Ha surfaces. The Machined (Figure 7- A, B, and C) and Nano-Ha (Figure 7- D, E, and F) surfaces showed greater amounts of live bacteria when compared to BSF18 (Figure 7- G, H, and I); this is possible to detect through visible results (Figure 7). BSF18 showed a mixture of dead and live bacteria when compared to the other groups. The thickness of the biofilm showed that Nano-Ha samples had a denser presence of live bacteria. Figure 8-A shows the 3D morphology of cells between groups from different surfaces.

The results of counting of live an dead analyzed by ImageJ software demonstrated a greater number for live cells for Nano-Ha surface when compared to machined and BSF18 (26.38±4.47; 7.85±0.84 and 2.65±1.04, respectively; p<0.05) and for dead cells, BSF18 showed increased value number when compared to machined and Nano-Ha groups (20.58±2.22; 0.90±0.12 and 2.47±0.87, respectively; p<0.05) (Figure 8-B). 

**Figure 7:**
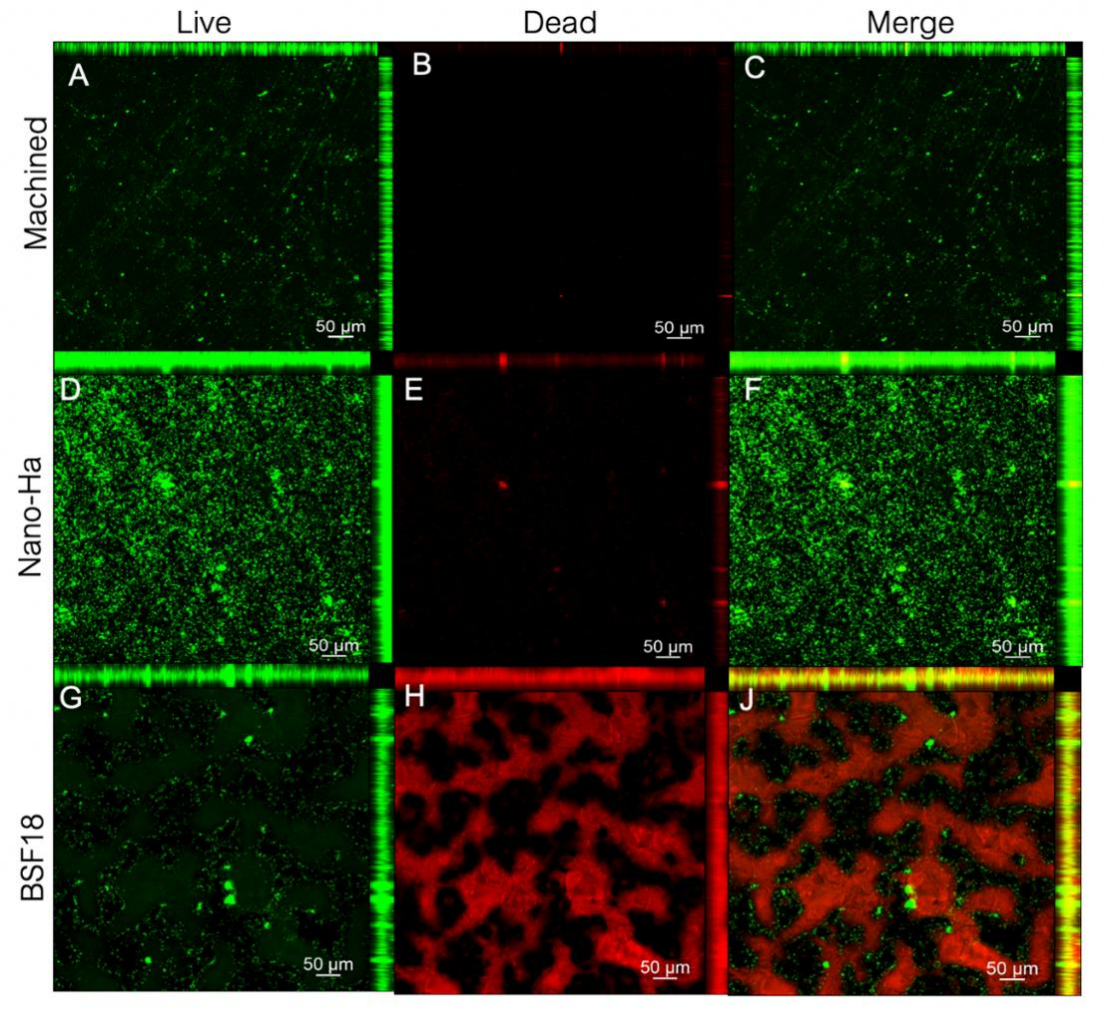
After 24 hours of cell cultivation, biofilm growth differed between the groups. A, B, C: Machined*.* D, E, and F: Nano-Ha. G, H, and I: BSF18. The cells were stained with LIVE/DEAD Biofilm Viability Kit Dye. Scale: 50 µm. The green color indicates live cells, and the red color indicates dead cells.

**Figure 8:**
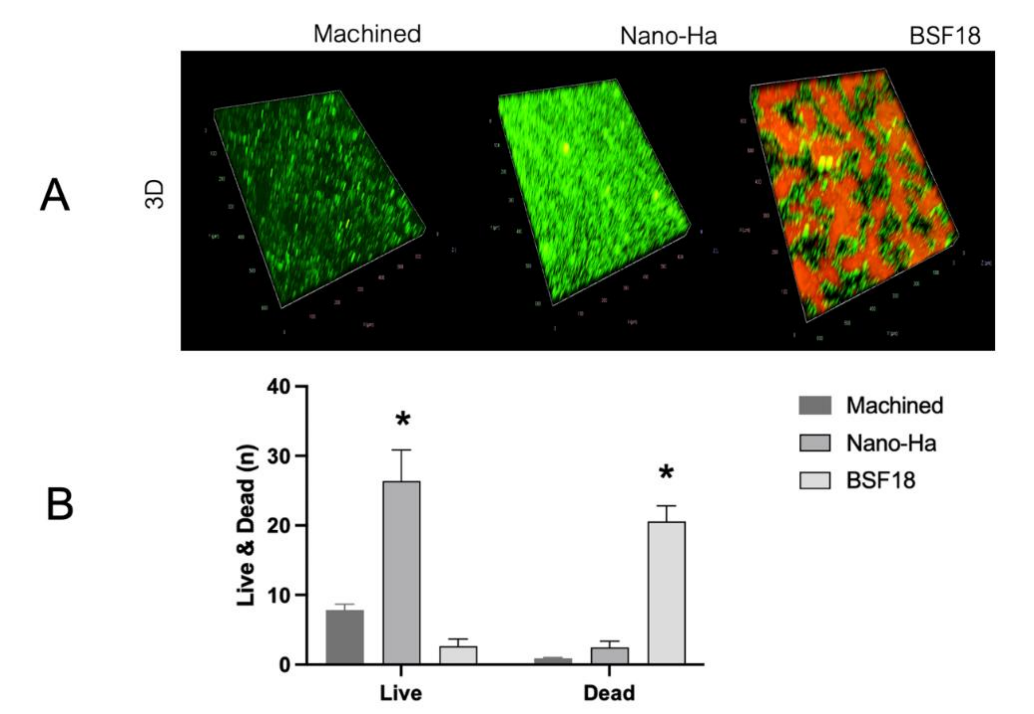
3D Images from confocal laser scanning microscopy analysis. **A.** Machined surface; Nano-Ha surface and BSF18 Surface. The cells were stained with LIVE/DEAD Biofilm Viability Kit Dye. Scale: 50 µm. The green color indicates live cells, and the red color indicates dead cells. B. The results of counting (n) of live and dead analyzed by ImageJ software demonstrated a greater number of live cells for Nano-Ha surface when compared to machined and BSF18 (26.38±4.47; 7.85±0.84 and 2.65±1.04, respectively; p<0.05) and for dead cells, BSF18 showed increased value number when compared to machined and Nano-Ha groups (20.58±2.22; 0.90±0.12 and 2.47±0.87, respectively; p<0.05). Two-way ANOVA test and Tukey’s comparisons

## Discussion

In the present study, titanium discs were coated with a novel bioactive glass composition (BSF18) and then used to evaluate anti-biofilm activity against *Staphylococcus aureus* biofilm growth after 24 hours. The graphs of disc surfaces indicated that only the BSF18 surface could induce the formation of the hydroxycarbonate apatite layer (HCA) after immersion for 24 hours in Simulated Body Fluid (SBF). The formation of a carbonated hydroxyapatite layer on the surface is described in all bioactive inorganic materials used for bone replacement. The HCA layer formation is a condition seen as necessary for the development of a chemical bond between bone and the biomaterial ^19^ ^,^ ^20^ ^,^ ^21^ ^,^ ^22^ ^,^ ^23^ ^,^ ^24^ ^,^ ^25^ ^,^ ^26^ ^,^ ^27^. A crucial factor in the osseointegration process is surface roughness, which can improve the process. A significant increase in surface roughness (Ra) was observed for Nano-HA and BSF18 discs in our study. According to the literature, these surfaces can be classified as moderately rough surfaces, and the machined samples can be classified as smooth surfaces ^28^ ^,^ ^29^. 

Regarding biofilm formation, the BSF18 samples had lower values of CFU/mL when compared to Machined and Nano-Ha samples. The values can be associated with SEM and fluorescence microscope representative biofilm images and values. The BSF18 discs had a smaller area coated with live cells; these findings can be correlated to the ones reported by Marques and co-authors ^19^, which showed that BSF18 surfaces demonstrated lesser contamination after periods of 2, 4, and 8 hours. A previous study investigated the coating of BSF18 on steel samples and Staphylococcus aureus and showed that F18 has anti-biofilm activity able to reduce more than 6 logs of the viable cells for 24 hours at a concentration of 50mg/mL^18^. In another study, it was demonstrated that F18 can be efficient for other pathogenic strains as well, such as *Staphylococcus aureus*, *Staphylococcus epidermidis*, *Escherichia coli,* and *Pseudomonas aeruginosa* ^17^. 

The antimicrobial mechanisms associated with the F18 bioactive glass surface can be partially explained by the confocal microscopy findings, which showed a high proportion of red-stained cells, indicating loss of bacterial membrane integrity. This observation suggests that membrane disruption may play a key role as a bactericidal mechanism. Studies indicate that the bacteriostatic and/or bactericidal action of bioactive glass (BAG) is associated with various mechanisms, such as pH elevation, increased osmotic pressure, and the presence of glass fragments, which can damage the cell walls of microorganisms, leading to bacterial inactivation ^30^ ^,^ ^31^. The antibacterial action in modern dental biomaterials is mainly achieved by bioactive glasses and ceramics that release ions and raise pH levels, damaging bacterial membranes, metabolism, and DNA, while often promoting bone regeneration. Silver, copper, niobium, and other elements can significantly enhance this effect when dosed optimally ^32^ ^,^ ^33^ ^,^ ^34^. These multifunctional coatings and frameworks demonstrate strong antibacterial performance in vitro and promising results in early-stage in vivo studies, but long-term clinical data and dosage safety windows remain active research needs.

In our study, we found that the biofunctionalization of titanium with F18 bioactive glass could reduce the number of bacteria attached to the sample surface when compared to other surface treatments (Machined and Nano-Ha). The advantage of coating titanium surfaces with F18 is that the thermal expansion coefficient of the glass matches the metal, and it prevents the glass from pulling away from the metal during the processing ^11^.

A previous study investigated an F18-functionalized dental implant on the mandible of dogs, reporting that, in a pre-clinical application, it showed better osseointegration (bone-implant-contact) for 2 weeks when compared to the control group, which means a clinically relevant effect on osseointegration at the early stages of healing ^22^. In Implant Dentistry, microbial colonization and biofilm formation on implant surfaces are influenced by the physicochemical properties ^3^ ^,^ ^4^, and the literature is investigating new coatings of implants for anti-microbial function, for example, bioglass. Alternative bioglass compositions have been investigated; however, changes in chemical composition increase bioactive glass crystallization, which negatively affects surface reactions and reduces bioactivity ^35^. 

The mechanism of action of bioactive glass in reducing microorganisms is still being investigated. Different coating technologies have been proposed for dental implants to improve the physical and chemical properties of bioglass coating in dental implant ^36^. Our current patented method of coating metal samples already produced on an industrial scale in a cost-efficient way is of potential interest. 

Bioglass F18 has a larger working range, which means it does not crystallize during processing while retaining very high bioactivity. It can be shown through high bioactivity, as shown in the formation of the hydroxycarbonate apatite (HCA) layer in 24 hours, so this phenomenon leads to a fast bone bonding mechanism. The contribution to osteogenic properties, F18, is also shown for potential anti-biofilm activity. The findings reported by Abushahba et al. 2024 ^37^ showed that bioactive glass (BG) applied through air particle abrasion (APA) can alter sandblasted and acid-etched titanium (SA Ti) surfaces by inducing the deposition of a calcium phosphate layer. In addition, this surface modification contributed to lower roughness values, increased surface wettability, and enhanced osteoblastic cell adhesion and survival. Another study demonstrated that bioglass coatings exhibited favorable mechanical and biological properties, which were directly influenced by the material’s composition. Among the groups evaluated, the 62W composition yielded the best results, with greater adhesion, apatite formation, and a better osteoblastic response ^38^. Furthermore, previous studies have already demonstrated that bioglass has been used as an implant coating even before hydroxyapatite, promoting greater bioactivity, increased metabolic activity of osteoblasts, bone regeneration, and antibacterial properties. Thus, bioglass proves to be a promising and viable alternative for stimulating bone formation and promoting better clinical outcomes in bone repair and implant dentistry applications ^39^ ^,^ ^40^ ^,^ ^41^.

Within the scope of this study, the biofunctionalization of titanium discs with bioactive glass F18 effectively inhibited the growth of Staphylococcus aureus biofilm after direct contact for 24 hours, resulting in reduced bacterial adhesion. However, it is important to interpret the findings cautiously due to significant limitations, such as the use of a monospecies biofilm model, a short experimental duration, and methodological constraints like the absence of ion release, pH variation, and broader biological assessments. Moreover, oral peri-implant biofilms are complex and multispecies, which could impact the antimicrobial efficacy of the material in clinical settings.

Despite these limitations, bioactive glass F18 exhibited promising antibacterial and bioactive properties, suggesting its potential as a synthetic biomaterial for combating Staphylococcus aureus biofilm. Therefore, it may serve as a viable option for future strategies targeting peri-implant disease prevention. Nonetheless, further in vitro and in vivo studies utilizing multispecies biofilm models and longer observation periods are essential to validate its clinical utility.

## Data Availability

The research data are available within the article
